# Incidence of Urinary Tract Infection After Combined Cystoscopy and Urodynamic Testing: A Prospective Cohort Study

**DOI:** 10.1002/nau.70244

**Published:** 2026-02-13

**Authors:** Camilo Andres Torres Sandoval, María Juliana Arcila, Andrés Duarte Osorio, Juan Guillermo Cataño, Sebastian Peña Rodriguez

**Affiliations:** ^1^ Department of Urology, Faculty of Medicine Pontificia Universidad Javeriana, Hospital Universitario San Ignacio Bogotá Colombia; ^2^ Department of Urology Hospital Universitario San Ignacio Bogotá Colombia; ^3^ Department of Preventive and Social Medicine, Faculty of Medicine Pontificia Universidad Javeriana Bogotá Colombia; ^4^ Faculty of Medicine Universidad de los Andes Bogotá Colombia

**Keywords:** cystoscopy, urinary tract infection, urodynamics

## Abstract

**Objective:**

To evaluate the incidence of urinary tract infection (UTI) after combined cystoscopy and urodynamic testing.

**Methods:**

A prospective analytical cohort study was conducted including adult patients undergoing cystoscopy, urodynamic testing, or both procedures during the same session at a quaternary referral center between May 1, 2023, and April 30, 2024. All patients were managed under an institution‐wide, urine culture‐guided antibiotic prophylaxis protocol. The cumulative incidence of UTI within 30 days after the procedure was calculated for the three cohorts. Sociodemographic characteristics, urodynamic parameters, and cystoscopic findings were analyzed to assess their association with UTI occurrence.

**Results:**

A total of 560 patients were included, of whom 473 (84.5%) completed follow‐up. Women accounted for 39.7% and men for 60.3% of the cohort, with a median age of 69 years. Seventeen patients underwent combined cystoscopy and urodynamic testing, 194 underwent urodynamic testing alone, and 262 underwent cystoscopy alone. No UTIs were observed in the combined cohort. The incidence of UTI was 2.6% following urodynamic testing and 1.9% following cystoscopy alone. No significant associations were identified between patient characteristics, urodynamic variables, or cystoscopic findings and the development of UTI.

**Conclusions:**

Combined cystoscopy and urodynamic testing do not appear to increase the risk of UTI compared with either procedure performed separately. Larger studies are required to confirm these findings and to define optimal antibiotic prophylaxis strategies and identify high‐risk subgroups.

## 1 Introduction

Urodynamic testing and cystoscopy are among the most commonly performed diagnostic procedures in urological practice. There is a growing tendency to perform both procedures during the same clinical session. The complications most frequently associated with these procedures include urinary tract infection (UTI), sepsis, hematuria, urethral trauma, bladder injury, and urethral stricture, among others. UTI is one of the most extensively studied complications, as it represents a nosocomial condition with significant public health implications due to its associated healthcare costs and the increasing risk of infections caused by multidrug‐resistant organisms. The reported incidence of UTI ranges from 1% to 21% following cystoscopy [1, 2, 3, 4] and from 3% to 28% following urodynamic testing [4, 5, 6, 7, 8]. Several risk factors have been described, including age, sex, smoking status, diabetes, use of immunosuppressive medications, and specific urodynamic parameters such as postvoid residual volume and maximum urinary flow rate (Qmax).

However, existing studies have primarily evaluated the risk of UTI associated with cystoscopy and urodynamic testing when each procedure is performed independently. Data regarding the incidence of UTI and associated risk factors when both procedures are performed concomitantly during the same session are limited. Therefore, the aim of this study was to determine the 30‐day cumulative incidence of UTI in patients undergoing combined cystoscopy and urodynamic testing and to identify factors associated with the development of this complication.

## 2 Materials and Methods

### 2.1 Study Objectives

The primary objective of this study was to determine the incidence of urinary tract infection (UTI) in a cohort of patients undergoing sequential cystoscopy and urodynamic testing during the same clinical session.

Secondary objectives included: (1) describing the incidence of UTI after cystoscopy alone; (2) describing the incidence of UTI after urodynamic testing alone; (3) identifying factors associated with the development of UTI in each cohort; and (4) describing microbial isolation patterns and antimicrobial resistance among patients who developed UTI.

### 2.2 Study Design and Setting

A prospective cohort study was conducted including adult men and women (≥ 18 years) who underwent cystoscopy, urodynamic testing, or both procedures during the same session at a quaternary referral center in Bogotá, Colombia, between May 1, 2023, and April 30, 2024. The indication for these procedures was determined by the treating physicians and not by the study investigators. Accordingly, the study did not intervene in clinical decision‐making and was limited to monitoring the incidence of urinary tract infection following the performance of these diagnostic procedures. Written informed consent for the procedures was obtained from all participants, and verbal informed consent was obtained for the follow‐up telephone call.

### 2.3 Procedures

Rigid cystoscopes with 17.5–19 Fr sheaths and a 30‐degree lens were used for cystoscopic examinations. Urodynamic testing was performed using Laborie urodynamic software according to institutional standards. All patients provided written informed consent prior to undergoing each diagnostic procedure in accordance with institutional protocols. Additionally, verbal informed consent was obtained from all participants for inclusion in the study and for the collection and analysis of clinical data.

### 2.4 Eligibility Criteria

Patients with permanent indwelling urinary catheters, those performing clean intermittent catheterization, patients with urinary diversions (ileal conduit, neobladder, or suprapubic cystostomy), patients requiring urinary catheter placement after the procedure, or those attending for double‐J stent removal were excluded.

### 2.5 Antibiotic Prophylaxis and Follow‐Up

According to institutional protocol, a pre‐procedural urine culture was required for all three procedures. Patients with a negative urine culture underwent the procedure without antibiotic prophylaxis. In cases of positive urine culture, targeted antibiotic prophylaxis was administered on the day of the procedure based on infectious disease specialist recommendations.

At the end of the procedure, patients who agreed received an order for a follow‐up urine culture within 30 days to assess the incidence of asymptomatic bacteriuria. All patients were provided with standardized warning signs prompting emergency consultation. Additionally, patients were contacted by phone call by urology residents or nursing staff within 30 days after the procedure to assess the presence of irritative urinary symptoms. For patients without available institutional medical records, relevant medical history was obtained during this contact.

If urinary symptoms suggestive of UTI were identified, patients were asked whether they had sought medical care or received antibiotic treatment either within or outside the institution. In cases of symptomatic UTI without prior medical evaluation, a urine culture was ordered and antibiotic treatment was initiated according to institutional guidelines. This approach is part of routine clinical care at the Urology Unit.

### 2.6 Outcome Definition

UTI was defined as the presence of irritative urinary symptoms accompanied by a positive urine culture and/or urinary symptoms that led to emergency department or outpatient consultation requiring antibiotic treatment.

### 2.7 Variables and Data Collection

Quantitative variables were summarized as means with standard deviations for normally distributed data or medians with interquartile ranges for non‐normally distributed data. Qualitative variables were summarized using absolute and relative frequencies.

### 2.8 Statistical Analysis

The cumulative incidence of UTI within 30 days after the procedure was calculated. Associations between sociodemographic variables, urodynamic parameters, cystoscopic findings, and UTI occurrence were evaluated separately for each cohort. Fisher's exact test was used for categorical variables, and the Mann–Whitney U test (Wilcoxon) was applied to continuous variables according to data distribution. Data were stored using Microsoft Excel and REDCap and analyzed using Jamovi software (version 2.3.21).

### 2.9 Use of Artificial Intelligence

Artificial intelligence tools were used to support the preparation of this manuscript. Specifically, ChatGPT (version 5.2; OpenAI, San Francisco, CA, USA) was utilized for language translation, writing assistance, and grammatical editing. The authors reviewed, edited, and take full responsibility for the content, accuracy, and integrity of the final manuscript.

## 3 Results

### 3.1 Study Population

A total of 560 patients met the inclusion criteria. Of these, 473 (84.5%) completed the scheduled telephone follow‐up. Women accounted for 39.7% (188/473) and men for 60.3% (285/473) of the population, with a median age of 69 years. Regarding the procedures performed, 17 patients underwent sequential cystoscopy and urodynamic testing during the same session (Cohort A), 194 underwent urodynamic testing alone (Cohort B), and 262 underwent cystoscopy alone (Cohort C).

Prior to the procedures, asymptomatic bacteriuria was identified in 7.6% (36/473) of patients. *Escherichia coli* was the most frequently isolated microorganism (57.9%, 22/38), followed by *Enterococcus faecalis* (15.8%, 6/38). Two patients had polymicrobial cultures; therefore, a total of 38 microorganisms were identified. Overall, 63% (26/38) of isolates were susceptible to all tested antibiotics, 13% (5/38) showed an extended‐spectrum beta‐lactamase (ESBL) pattern, and 7.8% (3/38) were resistant to fluoroquinolones. Table 1 illustrates all microorganisms isolated and their antimicrobial susceptibility patterns.

**Table 1:** Microorganisms isolated prior to the procedure.

	Procedure			
Microorganisms	Global *n* = 38 *n* (%)	UDS *n* = 10 *n* (%)	Cystoscopy *n* = 26 *n* (%)	UDS + Cystoscopy *n* = 2 *n* (%)
*E. coli*	22 (57.9)	7 (70)	14 (53.8)	1 (50)
*E. Faecalis*	6 (15.8)	0 (0)	6 (23.1)	0 (0)m
*K. pneumoniae*	2 (5.3)	0 (0)	1 (3.8)	1 (50)
*S. marcescens*	1 (2.6)	0 (0)	1 (3.8)	0 (0)
*M. morgagni*	1 (2.6)	0 (0)	1 (3.8)	0 (0)
*S. agalactiae*	1 (2.6)	0 (0)	1 (3.8)	0 (0)
*E. faecium*	1 (2.6)	0 (0)	1 (3.8)	0 (0)
*P. rettgeri*	1 (2.6)	0 (0)	1 (3.8)	0 (0)
*S. aureus*	1 (2.6)	1 (10)	0 (o)	0 (0)
*S. epidermidis*	1 (2.6)	1 (10)	0 (0)	0 (0)
recorded	2 (5,3)	1 (10)	1 (3.8)	0 (0)

Pattern	Global *n* = 38 *n* (%)	UDS *n* = 10 *n* (%)	Cystoscopy *n* = 26 *n* (%)	UDS + Cystoscopy *n* = 2 *n* (%)
Susceptible to all tested antibiotics	25 (65.7)	7 (70)	16 (61.5)	2 (100)
ESBL	5 (13.2)	0 (0)	5 (19.2)	0 (0)
Quinolones resistance	3 (7.9)	1 (10)	2 (7.7)	0 (0)
First generation Cephalosporine Resistance	1 (2.6)	0 (0)	1 (3.8)	0 (0)
AmpC	1 (2.6)	0 (0)	1 (3.8)	0 (0)
TMP/SMX resistance	1 (2.6)	1 (10)	0 (0)	0 (0)
Not described	2 (5,3)	1 (10)	1 (3.8)	0 (0)

Abbreviations: ESBL, Extended‐spectrum beta‐lactamase; TMP/SMX, trimethoprim/sulfamethoxazole.

### 3.2 Cohort A: Combined Cystoscopy and Urodynamic Testing

Among the 17 patients who underwent combined procedures, 12 were men and 5 were women. The most frequent indication in men was mixed lower urinary tract symptoms (6/12) and urinary incontinence (6/12), whereas in women the most common indication was urinary incontinence (3/5). Sociodemographic characteristics of this cohort are summarized in Table 2.

**Table 2:** Sociodemographic characteristics of patients undergoing combined cystoscopy and urodynamic testing.

Age, median (IQR)	70 (63–74)
Men, *n* (%)	12 (70,6)
Asymptomatic bacteriuria prior to the procedure, *n* (%)	2 (11,8)
**Procedure indication** *n* (%)	
Urinary incontinence	9 (52,9)
Mixed lower urinary tract symptoms	6 (35,2)
Hematuria + storage urinary symptoms	1 (5,9)
Recurrent urinary tract infection	1 (5,9)
**Past medical history** *n* (%)	
Diabetes Mellitus	5 (29,5)
Smoking	2 (11,8)
Use of immunosuppressive drugs	1 (5,9)
History of immunosuppression	4 (23,5)
Cancer	11 (64,7)
Genitourinary tract cancer	9 (52,9)

In 13 patients, cystoscopy was performed before urodynamic testing, while in the remaining four patients the order was reversed. Two patients (11.7%) had a positive pre‐procedural urine culture. The isolated microorganisms were *E. coli* (*n* = 1) and *Klebsiella pneumoniae* (*n* = 1), both susceptible to all tested antibiotics.

No episodes of urinary tract infection were observed during the follow‐up period. Urodynamic findings for this cohort are presented in Table 3. Regarding cystoscopic findings, no indirect signs of bladder outlet obstruction in women, such as urethral stricture or severe trabeculation, were reported. Cystoscopic findings in men are described in Table 4.

**Table 3:** Urodynamic characteristics of patients undergoing combined procedures.

	Men *n* = 12 Median (IQR)	Women *n* = 5 Median (IQR)
Bladder Maximum capacity	400 (240‐470)	325 (130–360)
Q max	7,5 (7–11,3)	8,5 (3,75–13,8)
pDet Qmax	35,5 (26,3–56,8)	18 (15,8–25,8)
BCI	82 (63,5–107)	69 (55–82,5)
BOOI	18(10–18,8)	N/A
fBOOI	N/A	8,2 ((−4,2) to 23,8)
PVR in flow–pressure (mL)	80 (48,8–119)	34 (24,8–91,3)
PVR in flow‐pressure > 100, n (%)	5 (41,6)	1 (20)

Abbreviations: N/A, Not applicable; PVR, Post‐void residual; BCI, Bladder contraction index; BOOI, Bladder Outlet Obstruction Index; fBOOI, Female Bladder Outlet Obstruction Index; pDet Qmax, Detrusor pressure at Qmax; Qmax, Maximum flow rate.

**Table 4:** Cystoscopic findings in men undergoing combined cystoscopy and urodynamic testing.

Variables	Findings	n (%)
Penile urethral stricture	No	11 (91,7)
	Yes	1 (8,3)
Bulbar urethral stricture	No	8 (66,7)
	≤ 50%	1 (8,3)
	50%–75%	2 (16,7)
	> 75%	1 (8,3)
Prostate	Bilobular	2 (16,7)
	Trilobular	2 (16,7)
	Wide prostatic cavity (history of TURP)	2 (16,7)
	History of radical prostatectomy	5 (41,7)
	Not visualized due to anterior urethral stricture	1 (8,3)
Verumontanum–bladder neck distance (VBN distance)	< 2.5 cm	4 (33.3)
	Not measured^a^	8 (66,7)

a Patients in whom the measurement was not performed due to a history of radical prostatectomy, TURP, or urethral stricture were included.

### 3.3 Cohort B: Urodynamic Testing

Of the 194 patients in this cohort, 59 were men and 135 were women. The most common indication for urodynamic testing was urinary incontinence, present in 65.4% of cases. Among men, the most frequent indication was mixed lower urinary tract symptoms (76.3%, 45/59), whereas among women it was urinary incontinence (84.4%, 114/135).

The incidence of UTI following urodynamic testing was 2.6% (5/194). Among patients with UTI, women accounted for 80% of cases, while men accounted for 20%. In two cases, UTI was confirmed by a positive urine culture, identifying *K. pneumoniae* (*n* = 1) and *E. faecalis* (*n* = 1), both susceptible to all tested antibiotics. In the remaining cases, urine cultures were not available, and UTI was defined based on the presence of urinary symptoms requiring antibiotic treatment. All of these patients sought emergency care at external institutions, and medical records were not accessible. Notably, all five patients had a negative pre‐procedural urine culture.

The most common indication for urodynamic testing among patients who developed UTI was evaluation of lower urinary tract symptoms (3/5), followed by urinary incontinence (2/5). Baseline characteristics of patients with and without UTI are presented in Table 5. The association between urodynamic findings in men and women and the occurrence of UTI is shown in Table 6.

**Table 5:** Baseline characteristics of patients with and without UTI after urodynamic testing.

	No UTI (*n* = 189) *n* (%)	UTI (*n* = 5) *n* (%)
Age^a^	64 (54–74)	72 (63–80)
Women	131 (69,3)	4 (80)
Men	58 (30,7)	1 (20)
Asymptomatic bacteriuria prior to the procedure, *n* (%)	10 (5,3)	0 (0)

Procedure indication	*n* (%)	*n* (%)
Urinary incontinence	137 (72.5)	2 (40)
Lower urinary tract symptoms		
Voiding symptoms	28 (14.8)	1 (20)^b^
Storage symptoms	19 (10)	1 (20)
Mixed symptoms	21 (11.1)	1 (20)
Recurrent UTI	4 (2.12)	0 (0)
Pelvic organ prolapse without urinary symptoms	1 (0.53)	0 (0)

Past medical history	*n* = 176 (%)^c^	*n* (%)
Diabetes Mellitus	34 (19.3)	0 (0)
Smoking	1 (0.6)	0
Use of immunosuppressive drugs	11 (46.5)	1 (20)
History of immunosuppression	23 (13)	0 (0)
Cancer	44 (25)	1 (20)
Genitourinary tract cancer	14 (8)	0 (0)

*Note:* There were no statistically significant differences between the UTI and non‐UTI groups for any of the variables (*p* > 0.05).

^a^ Data are presented as median and interquartile range.

^b^ This patient had a grade IV anterior pelvic organ prolapse.

^c^ Thirteen patients did not provide information on medical history either in person or by telephone and had no available hospital medical records to assess these variables.

**Table 6:** Urodynamic findings.

Urodynamic variables	No UTI *n* = 189 Median (IQR)	UTI *n* = 5 Median (IQR)
Bladder Maximum capacity	420 (310–500)	340 (330–400)
Q max	15 (8–22)	8 (7–12)
pDet‐Qmax	30 (23–42,8)	30 (24–35)
BCI	84,5 (60,8–115)	89 (71,3–106)
BOOI	25 (11–40)	31 **(N/A)** a
fBOOI	14,7 ((−18) to 35,2)	6,15 (−18,7 to 12,6)
PVR in flow–pressure (ml)	10 (0–130)	60 (0–113)
PVR in flow‐pressure > 100, *n* (%)	50 (26,5)	2 (40)

*Note:* There were no statistically significant differences between the UTI and non‐UTI groups for any of the variables (*p* > 0.05).

Abbreviations: N/A, Not applicable; PVR, Post‐void residual; BCI, Bladder contraction index; BOOI, Bladder Outlet Obstruction Index; fBOOI, Female Bladder Outlet Obstruction Index; pDet Qmax, Detrusor pressure at Qmax; Qmax, Maximum flow rate.

^a^ There is no interquartile range for the BOOI variable in the UTI group, as there was only one male patient.

### 3.4 Cohort C: Cystoscopy

Of the 262 patients who underwent cystoscopy, 214 were men and 48 were women. The most common indication for the procedure in both sexes was surveillance for bladder cancer, reported in 97 cases (77 men and 20 women), followed by hematuria (41 cases) and lower urinary tract symptoms (30 cases).

After the procedure, five patients (1.9%) met the definition of UTI; all were men. In one case, UTI was confirmed by a positive urine culture despite a negative pre‐procedural culture. The isolated microorganism was *E. coli*, resistant to trimethoprim/sulfamethoxazole but susceptible to other antibiotics. The remaining four cases were defined as UTI based on urinary symptoms requiring antibiotic treatment without confirmatory urine culture. Among these four patients, three had a negative pre‐procedural urine culture, and one had a positive pre‐procedural culture with *Enterococcus faecium* susceptible to all tested antibiotics.

The most common indication for cystoscopy among patients who developed UTI was hematuria (three cases), followed by bladder cancer surveillance (*n* = 1) and lower urinary tract symptoms (*n* = 1). Baseline characteristics of patients with UTI are detailed in Table 7. The association between cystoscopic findings in men and the risk of UTI is presented in Table 8.

**Table 7:** Baseline characteristics of patients with and without UTI after cystoscopy.

	No UTI (*n* = 257) *n* (%)	UTI (*n* = 5) *n* (%)
Age, median (IQR)	71 (64–77)	75 (67–83)
Women	48 (18,7)	0 (0)
Men	209 (81,3)	5 (100)
Asymptomatic bacteriuria prior to the procedure, *n* (%)	23 (8,9)	1 (20)

Procedure indication	*n* (%)	*n* (%)
Urothelial carcinoma	106 (41.2)	1 (20)
Lower urinary tract symptoms	67 (26,1)	1 (20)
Hematuria	41 (15,9)	3 (60)
Urinary incontinence	22 (8.6)	0 (0)
Urethral stricture	16 (0)	0 (0)
Abnormal findings on imaging studies	7 (2.7)	0 (0)
Recurrent UTI	5 (1,9)	0 (0)
Colovesical fistula	1 (0.39)	0 (0)

Past medical history	*n* (%)	*n* (%)
Diabetes Mellitus^a^	55/253 (21.7)	2/5 (40)
Smoking^a^	12/253 (4,7)	0/5 (0)
Use of immunosuppressive drugs^a^	20/253 (7,9)	1/5 (20)
History of immunosuppression^a^	27/253 (10,6)	2/5 (40)
Cancer^b^	197/254 (77.5)	3/5 (60)
Urological cancer^b^	170/254 (67)	3/5 (60)

*Note:* There were no statistically significant differences between the UTI and non‐UTI groups for any of the variables (*p* > 0.05).

^a^ These variables were assessed in 258/262 patients; four patients did not provide medical history information either in person or by telephone and had no available hospital medical records.

^b^ This variable was assessed in 259/262 patients; three patients did not provide medical history information either in person or by telephone and had no available hospital medical records to assess this variable.

**Table 8:** Cystoscopic findings in patients with and without UTI.

Variables	Findings	Global (*n* = 214) *n* (%)	No UTI (*n* = 209) *n* (%)	UTI (*n* = 5) *n* (%)
Penile urethral stricture	No	191 (89.3)	186 (88.9)	5 (100)
	yes	23 (10.7)	23 (11.1)	0 (0)
Bulbar urethral stricture	No	162 (75,7)	159 (76,1)	3 (60)
	< 50%	17 (7,9)	17 (8,1)	0 (0)
	50%–75%	14 (6,5)	13 (6,2)	1 (20)
	> 75%	21 (9,8)	20 (9,6)	1 (20)
Prostate	Bilobular	81 (37.8)	78 (37.3)	3 (60)
	Trilobular	57 (26,6)	56 (26,7)	1 (20)
	Wide prostatic cavity (history of TURP)	14 (6,5)	14 (6,7)	0 (0)
	History of radical prostatectomy	48 (22.4)	48 (22.9)	0 (0)
	Not visualized or measured	14 (6.5.)	13 (6.2)	1 (20)
Verumontanum–bladder neck distance (VBN distance)	≤ 2.5	91 (42.5)	87 (41.6)	4 (80)
	> 2.5 cm	38 (17,8)	38 (18,2)	0 (0)
	Not measured^a^	85 (39.7)	84 (40.2)	1 (20)

*Note:* Patients in whom the prostate was not visualized due to penile or bulbar urethral stricture, and one patient in whom prostate morphology was not described and was only reported as non‐obstructive, were included. There were no statistically significant differences between the UTI and non‐UTI groups for any of the variables (*p* > 0.05).

^a^ Patients in whom the measurement was not performed. It also includes those patients in which the measurement was not possible due to a history of radical prostatectomy, TURP, or urethral stricture.

## 4 Discussion

The European Association of Urology (EAU) guidelines on urological infections (2025) provide a strong recommendation against the routine use of antibiotic prophylaxis for cystoscopy and urodynamic testing [9]. Similarly, the American Urological Association (AUA) states that antibiotic prophylaxis is not indicated for these procedures in patients without risk factors, given their low associated risk of urinary tract infection (UTI) [10]. However, both guidelines address these recommendations separately for each procedure and do not specify whether additional measures should be considered when cystoscopy and urodynamic testing are performed during the same clinical session.

As a result, institutional protocols for these procedures vary widely. In some centers, both studies are performed without requiring a pre‐procedural urine culture, whereas in others, urine culture–guided antibiotic prophylaxis is administered. This variability underscores the need for studies evaluating the incidence of UTI and associated risk factors following cystoscopy and urodynamic testing, whether performed individually or concomitantly during the same session.

Patient‐related risk factors most consistently associated with post‐procedural UTI include advanced age (> 70 years), diabetes mellitus, immunosuppression, and the presence of bacteriuria [10, 11, 12, 13, 14, 15]. These factors were systematically evaluated in each cohort of the present study.

### 4.1 Cystoscopy Cohort

The incidence of urinary tract infection (UTI) following cystoscopy (1.9%) lies within the range reported in the literature (1%–21%) and is comparable to the incidence previously reported in Colombia by García‐Perdomo et al. (2013), who documented a rate of 1.8% [11]. Although that study was a randomized controlled trial that included a group receiving antibiotic prophylaxis, all participants had a negative pre‐procedural urine culture; consequently, the role of asymptomatic bacteriuria as a potential risk factor could not be evaluated.

In this cohort, the prevalence of pre‐procedural asymptomatic bacteriuria was 9% and was not associated with an increased risk of UTI. This finding is consistent with the results reported by Falkenrodt et al. (2025), who found no significant association between asymptomatic bacteriuria and subsequent UTI (OR 1.39; 95% CI 0.51–3.74; *p* = 0.52) [15]. However, in that study, specific indications for cystoscopy—such as evaluation for suspected urothelial carcinoma and urinary retention—were associated with a higher risk of infectious complications. In contrast, in the present study, indications such as bladder cancer surveillance and voiding symptoms, although among the most prevalent, were not associated with an increased risk of UTI.

Furthermore, no significant associations were identified between UTI occurrence and sex, age, history of immunosuppression, diabetes mellitus, endoscopic prostate size, or the presence of urethral stricture in men. These findings are consistent with those of a prospective study including 300 patients who underwent cystoscopy with or without antibiotic prophylaxis, in which diabetes, age, urethral stricture, and prostate enlargement were not associated with post‐procedural UTI [16]. It should be noted, however, that all patients in that study had a negative pre‐procedural urine culture, and no subgroup analyses were conducted according to stricture location or prostate size.

### 4.2 Urodynamic Testing Cohort

In the urodynamic testing cohort, the incidence of post‐procedural UTI (2.6%) was also within the range reported in the literature (3%–28%). Previous studies have identified postvoid residual volume > 100 mL and maximum urinary flow rate (Qmax) < 15 mL/s as risk factors for UTI [6, 12, 13, 14]. In our analysis, urodynamic variables—including bladder capacity, postvoid residual volume during pressure‐flow studies, BOOI, fBOOI, and BCI—were not associated with UTI occurrence in either men or women.

These findings are consistent with those reported by Benseler et al. (2022) [5] but differ from those of Fox et al. (2020) [12]. It is important to note that the latter study included videourodynamic testing and patients with neurogenic bladder, and did not exclude individuals performing intermittent self‐catheterization—an established risk factor for UTI. These populations were excluded from our study. Nadeem et al. (2017) reported that postvoid residual volume > 100 mL and Qmax < 15 mL/s were associated with UTI in a prospective cohort of 100 patients, with an overall UTI incidence of 12% [6]. The higher incidence observed in that study may be related to the fact that 63% of patients underwent urodynamic testing for evaluation of lower urinary tract symptoms. In our cohort, this indication accounted for a smaller proportion of the overall population (35%) but was the most prevalent indication among patients who developed UTI (60%).

Patient‐related variables such as sex, diabetes, chronic kidney disease, malignancy, and immunosuppression were not associated with UTI development, consistent with prior reports [11]. Active smoking could not be analyzed as a risk factor, as no patients with UTI reported current tobacco use. This finding may reflect selection bias, as patients with storage symptoms are routinely advised to cease smoking as part of their management, and these patients constitute a substantial proportion of those undergoing urodynamic testing.

### 4.3 Combined Cystoscopy and Urodynamic Testing Cohort

No UTIs were observed in patients undergoing combined cystoscopy and urodynamic testing, despite the presence of a relevant proportion of known risk factors, as shown in Tables 2, 3, 4. This finding may be explained by the small sample size and the inherently low incidence of UTI associated with each procedure when performed independently. In this context, the sequential performance of both studies does not appear to justify modifications to current antibiotic prophylaxis protocols. However, these results should be interpreted cautiously given the limited size of this cohort, which restricts the ability to detect statistically significant differences in rare events. Larger, multicenter studies are therefore required to validate these findings and strengthen the available evidence.

Additionally, the order in which the procedures were performed during the same session did not appear to influence infection rates. From a technical standpoint, initiating the session with cystoscopy may offer practical advantages, including ensuring adequate bladder filling for uroflowmetry, improved assessment of urethral anatomy facilitating safe catheter placement, better bladder visualization by avoiding catheter‐induced hematuria, and reduced overall study time. However, these potential benefits were not specifically evaluated in the present study and should be addressed in future research.

### 4.4 Asymptomatic Bacteriuria

The prevalence of pre‐procedural asymptomatic bacteriuria in our study was 7.6%, which falls within the 4%–14% range reported in the literature 12. Although asymptomatic bacteriuria was not associated with UTI development in any cohort, a urine culture‐guided antibiotic prophylaxis protocol was used. Comparative studies including patients with asymptomatic bacteriuria undergoing these procedures, with and without antibiotic coverage, are needed to determine whether differences in UTI incidence exist.

Considering the evidence presented in the EAU and AUA guidelines and our findings, we believe that cystoscopic or urodynamic findings alone should not be used as criteria to modify established antibiotic prophylaxis protocols. Moreover, when antibiotic prophylaxis is guided by urine culture results, low rates of UTI can be maintained whether these procedures are performed individually or concomitantly.

## 5 Limitations

This study has several limitations. First, the small number of patients undergoing combined cystoscopy and urodynamic testing limits the strength and generalizability of the conclusions. This may be attributed to concerns regarding infection risk, patient discomfort, and logistical challenges associated with scheduling both procedures on the same day. These hypotheses warrant further investigation.

Second, as a referral center, a proportion of patients did not receive follow‐up care within our institution. Although telephone follow‐up mitigated this limitation to some extent, some patients declined to provide information, resulting in missing data. Approximately 15% of the total sample lacked complete follow‐up information, which may have led to underestimation of UTI incidence or its association with studied variables.

Finally, the definition of UTI included symptomatic cases requiring antibiotic treatment even in the absence of confirmatory urine culture. While clinically relevant for capturing infections managed outside the institution, this broad definition may have overestimated the true incidence of UTI, particularly in cases where symptoms were attributable to post‐procedural irritation or noninfectious causes.

## 6 Conclusions

The concomitant performance of cystoscopy and urodynamic testing may not be associated with a higher risk of urinary tract infection compared with performing these procedures separately. Larger studies with greater sample sizes are required to validate these preliminary observations and to establish specific recommendations regarding the execution of both procedures during the same clinical session. In particular, there is a need to define clear criteria for antibiotic prophylaxis and to identify high‐risk subpopulations in whom deferring combined procedures may be more appropriate. Generating this evidence is essential to develop clear and consistent recommendations, optimize clinical practice, and avoid the indiscriminate use of antibiotics.

## Funding

This research did not receive any specific grant from funding agencies in the public, commercial, or not‐for‐profit sectors.

## Ethics Statement

This study was approved by the Ethics Committee of the Hospital Universitario San Ignacio and Pontificia Universidad Javeriana, Bogotá, Colombia. FM‐CIE‐1272‐22.

## Consent

Informed consent was obtained from all participants included in the study.

## Conflicts of Interest

The authors declare no conflicts of interest.

## Data Availability

The data that support the findings of this study are available from the corresponding author upon reasonable request.
